# Emergency Nurses' Perceptions and Experiences in Managing Acute Pain in Critically Ill Adult Patients: A Qualitative Study

**DOI:** 10.1111/jan.17033

**Published:** 2025-05-10

**Authors:** Wayne Varndell, Margaret Fry, Doug Elliott

**Affiliations:** ^1^ Prince of Wales Hospital Emergency Department Randwick Australia; ^2^ Faculty of Health University of Technology Sydney Sydney Australia

**Keywords:** critically ill patient, emergency care, emergency nurse, non‐participant observation, pain

## Abstract

**Aim:**

The aim of this study was to examine the perceptions and experiences of emergency nurses managing acute pain in critically ill patients.

**Design:**

Qualitative descriptive study.

**Method:**

Non‐participant observations (*n* = 46, 157 h across 10 shifts) and semi‐structured interviews (*n* = 30) were conducted at two Australian metropolitan emergency departments from November to December 2020. Transcribed data were analysed using reflexive thematic analysis.

**Results:**

The qualitative analysis yielded three main themes and eight subthemes: (i) *being in the resuscitation area* in which participants detail learning to work in the resuscitation area; influences in managing critically ill patients and confidence in acute pain management; (ii) *prioritising pain management* identified the challenges in optimising pain management and balancing departmental demands; and (iii) *between being and doing* and how nurse–patient interactions and care behaviours impacted on optimising pain management and comfort in the resuscitation area.

**Conclusion:**

Emergency nurses were primarily responsible for the continuity of patient care and optimisation of pain control for critically ill patients. Confidence in managing acute pain in critically ill patients was variable. While nurses actively sought ways to provide a reassuring presence and comfort to critically ill patients, this was limited by unpredictable workloads, availability of staff and communication challenges.

**Implications for the Profession and/or Patient Care:**

These study findings may assist in the development of policy and formal education of emergency nurses transitioning into the resuscitation area and the management of acute pain in critically ill patients.

**Impact:**

Pain is under‐assessed and managed in critically ill patients, and this could stem from emergency nurses' practices. The findings could inform interventions to enhance pain management and practices.

**Patient or Public Contribution:**

No patient or public contribution.

**Reporting Method:**

This study adhered to the COREQ criteria.

## 1 Introduction

Emergency departments (ED) across Australia are increasingly pressured to meet rising demands for timely critical care, despite increasing patient access block to acute and critical care services (Richardson and Mountain 2009). Although the phrase *critical care* is often associated with intensive care units, this level of nursing care is increasingly provided to a wide range of patient groups for extended periods of time within a specifically designated resuscitation and trauma area (Chalfin et al. 2007). Adequate analgesia is paramount in optimising comfort (Reade and Finfer 2014), wellbeing, and safety of critically ill patients (College of Emergency Nursing Australasia 2011), who are often inflicted with a barrage of noxious stimuli and invasive procedures (e.g., insertion of intravenous and urinary catheters, chest drains, endotracheal intubation) in the course of resuscitation and stabilisation. Emergency nurses, as an extension to resuscitating and stabilising critically ill patients, are increasingly responsible for the continuity of patient care, which includes pain management. There is, however, little evidence within the international literature pertaining to how emergency nurses accomplish this.

### 1.1 Background

Pain is the most common presenting symptom to the ED worldwide (Todd et al. 2007; Häske et al. 2024; Cordell et al. 2002). In Australia, over 9 million patients present to the ED (Australian Institute of Health and Welfare 2025), with an 18.3% increase from 2014 to 2024 (*n* = 1,651,959) (Australian Institute of Health and Welfare 2015, 2025). Between 51% (Hughes et al. 2021) and 78.8% (Bureau of Health Information 2023) of all patient presentations to the ED are due to experiencing unrelieved acute pain. This equates to 4.60 and 7.11 million patients presenting with pain to the ED each year in Australia alone (Australian Institute of Health and Welfare 2025). Further, over the last decade, the number of patients presenting to ED with life‐threatening injuries or illness has nearly doubled (Australasian Triage Scale category 1 and 2, 44.6%, *n* = 718,873) (Australian Institute of Health and Welfare 2015, 2025; Australasian College for Emergency Medicine 2023). A third of whom (30.1%) require intubation and mechanical ventilation in ED prior to transfer to intensive care (Australian and New Zealand Intensive Care Society 2019). Many (40%) critically ill patients experience moderate to severe pain due to underlying illness or injury, or noxious stimuli caused by interventions during resuscitation and stabilisation (Wu et al. 2022). Unfortunately, pain is often poorly treated in the ED (Todd et al. 2007; Bijur et al. 2012; Ducharme and Barber 1995; Fosnocht et al. 2001; Guru and Dubinsky 2000; Lee et al. 2008). The consequences of unrelieved acute pain are increased cardiovascular, respiratory, coagulation and gastrointestinal complications, as well as increased neurohumoral stress response (Schug et al. 2015; Porth and Grossman 2018; Zhang et al. 2018) Traditionally, relief from pain through the administration of analgesics could only be initiated by physicians (Dewhirst et al. 2017). Since pain can affect the morbidity and mortality of critically ill patients (Hussien and Hay 2022), adequate relief is an essential part of providing emergency nursing care.

Emergency nurses, as frontline clinicians, are frequently in a position to assess for and optimise acute pain management as they have the most contact with the patient and their family members (Varndell et al. 2018). Emergency nurses play a vital role in the assessment and management of pain and discomfort in critically ill patients through analgesia, often using opioids, non‐opioid analgesics and non‐pharmacological methods, while carefully assessing and monitoring the patient's response (Lord and Varndell 2024). Different strategies have been developed in response to inadequate pain management, such as pain management protocols or clinical guidelines and educational interventions for emergency nurses (Burgess et al. 2021). While nurse‐initiated analgesia protocols have been implemented successfully to decrease the time to first dose of analgesics in the ED, they are largely focused on managing pain in low‐acuity patient populations (Varndell et al. 2018). There is little evidence of nurse‐initiated pain management protocols being implemented for critically ill patients in the ED (Varndell et al. 2015; Varndell et al. 2020). Investigating how emergency nurses assess and manage acute pain in critically ill patients in the resuscitation and trauma area is unknown and warranted.

### 1.2 Aim

This study examined perceptions and experiences of emergency nurses managing acute pain in critically ill patients cared for in the resuscitation area.

## 2 Methods

### 2.1 Study Design

This study used an explanatory sequential mixed‐methods approach, the protocol of which is described elsewhere (Varndell et al. 2017a). Data collection occurred in two sequential stages. Phase 1 used a quantitative cross‐sectional survey (*n* = 450) (Varndell et al. 2020) developed using a real‐time Delphi method to explore emergency nurses' practices in managing acute pain in critically ill patients, the processes and factors influencing nurses' acute pain management practices, and incorporated two validated questionnaires (Ferrell and McCaffery 2014; Guttormson et al. 2010). The cross‐sectional survey was conducted between 1st September and 31st October 2018. For context, a summary of phase 1 findings is provided in Table 1. This paper reports on the primary analysis of phase 2 data, which was delayed due to the COVID‐19 pandemic.

**TABLE 1:** Summary of phase 1 findings.

Training and education	Clinical experience (average 1–3 years) and workplace training required prior to using of analgesic standing orders. Infrequent training on assessment and management of acute pain in critically ill patients. Availability of ongoing workplace education and training in pain management was reported as poor
Practice	Majority (> 70%) of emergency nurses reported being able to initiate analgesia using local policy. Changes in vital signs commonly used to indicate the presence/relief of pain. A range of pain assessment instruments were used across EDs in Australia; few (< 10%) used instruments validated in the critically ill patient population
Pain management knowledge	The nurses' knowledge and attitudes about pain questionnaire (Ferrell and McCaffery 2014) found that the overall pain management knowledge of emergency nurses was low (< 75%). Weakest scoring knowledge areas included pharmacology, pain management strategies, addiction and spiritual/cultural beliefs
Factors influencing administration of analgesia	The nurse sedation practices survey (Guttormson et al. 2010) revealed emergency nurses had a strong supportive attitude towards using analgesia in critically ill patients, and was influenced by the frequency of education, level of knowledge and presence of senior nurses in the workplace. Ability to influence prescribing of analgesia, workload and availability of staff to assist had the greatest negative impact on administration of analgesia
Nurses' perception of pain	Across 26 common critical care activities (e.g., mechanical ventilation, endotracheal suctioning, indwelling catheter insertion), the majority (46.1%) were perceived as causing only mild levels pain (Visual Analogue Scale, 10–39 mm). However, a positive correlation was observed between years of nursing experience and the greater degree of perceived pain intensity

This qualitative study embedded within a constructivist paradigm builds upon phase 1 data from the above‐mentioned study with the objective of: (i) investigating the practices and behaviours of emergency nurses in the management of acute pain in critically ill patients in the resuscitation area; (ii) examining how emergency nurses influenced acute pain management; and (iii) identifying possible factors or barriers influencing acute pain management by emergency nurses. A qualitative descriptive approach based upon the work of Sandelowski (2010) was adopted and involved non‐participant observations and in‐depth semi‐structured interviews with emergency nurses working in the resuscitation area, leveraging the insights from the observations.

### 2.2 Study Setting and Recruitment

The non‐participant observations and semi‐structured interviews were conducted in two New South Wales metropolitan trauma‐designated EDs, each with a 2020 patient census of approximately 80,000, 18% of which were categorised as critically ill or injured (triage category 1 or 2) (Table 2). The study sites had 154 (Site 1) and 145 (Site 2) full time equivalent nurses, and three and five resuscitation bays, respectively. With the support of the ED leadership team, study information was distributed to emergency nurses at department meetings and posters were placed on staff notice boards. To limit the Hawthorne effect, the specific activity of pain management was not directly stated, but rather that the study would explore how emergency nurses cared for patients in the resuscitation area. A purposive sampling strategy was used to capture a range of clinical experience from emergency nurses for the data collection approaches. Eligible participants were nurses permanently employed in the ED with over 12 months experience working independently in the resuscitation area (Table 2).

**TABLE 2:** Total number of patient presentations at study sites for 2020 by triage category.

Triage category	Site 1	Site 2
1	685 (0.9)	1115 (1.4)
2	10,453 (13.1)	16,774 (20.7)
3	35,440 (44.3)	23,352 (28.8)
4	23,313 (29.1)	28,381 (35)
5	10,106 (12.6)	11,423 (14.1)
Total	79,997 (100.0)	81,045 (100.0)

In qualitative research, data saturation is not static but rather a dynamic process influenced by a variety of factors such as the research design and phenomena being investigated (Ahmed 2025). Non‐participant observations in the resuscitation area continued until data saturation was achieved and no new patterns or insights emerged. This was monitored by adopting a reflexive stance comprising journaling and ongoing critical discussions with the research team to maintain awareness of saturation progress, reducing bias and enhancing data richness (Olmos‐Vega et al. 2023). In exploratory studies involving participants with lived experience, 10–15 participants is sufficient to identify key themes and ensure the integrity and reliability of the data (Hennink and Kaiser 2022). A sample size of 15 participants per study site was considered appropriate for this study.

### 2.3 Data Collection

Non‐participant observations and semi‐structured interviews were conducted from November to December 2020, employing a qualitative descriptive approach to understanding emergency nurses' practices and experiences in managing acute pain in critically ill patients in the resuscitation area. The resuscitation and trauma area is a specially provisioned clinical space within the ED that provides reception, assessment, and the initial treatment for patients with life‐threatening illness or injury (Agency for Clinical Innovation 2024).

#### 2.3.1 Non‐Participant Observations

Non‐participant observations provided the opportunity to gain a more in‐depth understanding of the reality and agency of nursing practice in the resuscitation area; observing what nurses do in everyday practice (Eldh et al. 2020). For non‐participant observations, the primary author observed emergency nurses working in the resuscitation area managing critically ill patients. The work of Spradley (2016), LeCompte (2000) and Dewalt and Dewalt (2002) informed the development of an observation guide for field notes to interpret the flow of emergency nurses' interactions and behaviours in the resuscitation area relating to assessing and managing acute pain (Data S1).

To understand the participant's thought processes, particularly the outcomes of their pain management decision‐making processes, participants were encouraged to think out aloud (Aitken et al. 2011). Informal questioning was also used to clarify observations and to explore the cognitive processes behind nursing practices (Phillippi and Lauderdale 2017). Based on the literature, pain assessment should occur within 20 min of patient arrival and then re‐assessed at 60 min following administration of analgesia (European Society for Emergency Medicine 2020). Therefore, participants were observed for a minimum of 2 h. Activity relating to pain was defined as any interaction between nurse and patient or patient‐related communication or documentation that concerned the patient's pain or comfort. In consultation with the ED leadership team at each site, two observation times were identified as key periods for high activity in the resuscitation area. This covered change of shift, staff overlap times, and staff meal breaks. A pilot study was conducted with one participant to ensure that the observation guide was appropriate and consistent with the study's aims and objectives. Observations occurred from Monday to Friday between 07:00 and 12:30 h (*n* = 24, 52.2%) and 13:00 and 20:00 h (*n* = 22, 47.8%). A total of 157 h were spent observing emergency nurses (*n* = 46) for an average of 3.3 h (SD 0.7 h, range 2.1–4.4 h).

#### 2.3.2 Semi‐Structured Interviews

Semi‐structured interviews were conducted to expand upon observations made in the resuscitation area and to explore factors influencing and/or impacting upon emergency nurses' practice in detecting and optimising pain management in critically ill patients. The majority (*n* = 27, 90%) of interviews occurred immediately after the period of observation or were scheduled for when the participant was next on duty. Quinn's (2014) framework for semi‐structured interviews was used to guide the development of the interview schedule. This included questions exploring the participant's background, knowledge, sensory, behaviours/experiences, opinions/values and feelings/emotions (Data S2). The interview schedule began with demographics questions to help put participants at ease (Hinton and Ryan 2020), followed by 12 open‐ended questions and 4 self‐rating questions, exploring the clinical experiences of assessing, monitoring and managing acute pain in critically ill adult patients. Questions were structured to be open‐ended to enable participants to respond in their own words, and the freedom to describe their everyday experiences with as much detail as needed (Semyonov‐Tal and Lewin‐Epstein 2021). A pilot study was conducted with one participant to ensure that the interview guide was appropriate and consistent with the study's aims and objectives. All interviews were audio‐recorded and conducted at suitable locations to allow participants to return to the clinical floor if required. Interviews lasted on average 34 min (SD 6 min, range 21–44 min) and generated 158 pages of transcribed notes.

### 2.4 Data Analysis

Data from field notes (observations, artefacts and informal questions) and semi‐structured interviews formed the written elements of the qualitative dataset. Analysis of the data obtained were conducted in two stages: data preparation and thematic analysis. Data preparation was initiated by transcribing verbatim the field notes and audio‐recorded interviews into one coherent document and imported into NVivo (version 12) (QRS International Pty Ltd 2018). Field notes and interviews were analysed concurrently using an inductive and iterative process guided by the work of Braun and Clarke (2006). The first stage of thematic analysis is familiarisation with the data. Field notes and interviews were read by the authors (W.V. and M.F.) to become better acquainted and gain an overall understanding of the data, and initial notes were created. Second, a two‐step process was undertaken to generate initial codes. Textual data were segmented into smaller units: groups of words, sentences or paragraphs that contained particular aspects related to the study purposes (Graneheim and Lundman 2004). After that, each data segment were (re‐)read and coded according to the essence identified from the unit of the data, allowing data to be thought of in new and different ways (Ayton et al. 2023). During this stage, data were considered from a range of perspectives. Third, the initial codes were then developed into higher order sub‐themes according to a commonality or relationship within a group of codes (Graneheim and Lundman 2004). Using a reflexive approach allowed for refinement of the sub‐themes into main themes (Olmos‐Vega et al. 2023). Theme generation was supported by memo writing—a constant flow of free writing, reflections and notes (Charmaz 2014). Fourth, themes were reviewed by all authors to check whether they related to the coded extracts and to assure internal thematic coherence. Fifth, themes were defined and named. Finally, rich, thick descriptive narratives were provided to support the themes generated. This process assisted in increasing transferability, credibility and trustworthiness of qualitative research findings, allowing other researchers to assess their applicability to other contexts and settings (Younas et al. 2023).

#### 2.4.1 Ethical Considerations

This study phase was approved by the South Eastern Sydney Human Research and Ethics Committee (2020/ETH01114) on July 29th, 2020. An information sheet and consent form were provided to each participant. All identifiable features of the participants were coded to maintain confidentiality and privacy with site number (S1, Site 1 and S2, Site 2) and participant designation (RN, Registered Nurse; CNS, Clinical Nurse Specialist; CNE, Clinical Nurse Educator; CNC, Clinical Nurse Consultant) noted in parentheses only.

#### 2.4.2 Rigour and Reflexivity

Constructs for establishing trustworthiness and methodological rigour were based on Guba and Lincoln's framework (Ahmed 2024). Credibility of the research was strengthened by carefully describing how the analysis was conducted and by reporting the results following the categories of the analysis, with rich quotations and use of relevant tables and figures. Credibility, dependability, and transferability were achieved through dense description of the methodology used, and description of the data. Further, all interview materials, transcriptions, documents, findings, interpretations, and recommendations were kept available and accessible to the research team for the purpose of conducting an audit trail, and descriptions, codes, and themes were confirmed. Confirmability was demonstrated in two ways. First, during the informal questioning and interviews, the researcher restated and summarised information recorded to verify accuracy. Secondly, to reduce bias, the primary researcher adopted a reflexive stance whereby interpretations, personal beliefs, judgements, and practices were journaled and discussed with the research team who have extensive experience in qualitative research (Olmos‐Vega et al. 2023; McSweeney 2021).

## 3 Findings

### 3.1 Characteristics of Participants

The majority of participants (*n* = 35, 76.1%) were female with an average age of 37.3 years (SD 8.9, range 24–57), nearly 8 years of emergency nursing experience (mean 7.5, SD 2.9, range 3–15) and over 5 years' experience working in the resuscitation area (mean 5.2, SD 2.6, range 2–13). The majority held a postgraduate qualification in nursing (93.5%) (Table 3).

**TABLE 3:** Participant characteristics.

Characteristics	*N* (%)
Age, years (mean, SD, range)	37.3 (8.9, 24–57)
Sex	
Female	35 (76.1)
Male	11 (23.9)
Experience (mean, SD, range)	
Emergency nursing	7.5 (2.9, 3–15)
Working in the resuscitation area	5.2 (2.6, 2–13)
Nursing role	
Registered Nurse	28 (60.7)
Clinical Nurse Specialist	11 (23.9)
Clinical Nurse Educator	4 (8.7)
Clinical Nurse Consultant	3 (6.5)
Postgraduate qualification	43 (93.5)

Participants were observed providing care to 46 critically ill patients (Site 1: *n* = 22, 47.8%; Site 2: *n* = 24, 52.2%), who were typically male (*n* = 26; 56.5%), 51.2 years old (SD 19.1) with immediate life‐threatening emergencies (ATS 2; *n* = 38, 82.6%); traumatic injuries (*n* = 28, 60.8%), cardiac arrhythmias (*n* = 7, 15.2%), neurological (*n* = 4; 8.7%) or toxicology (*n* = 4, 8.7%) emergencies (Table 4).

**TABLE 4:** Characteristics of patients managed by participants.

Characteristics	*N* (%)
Age, years (mean, SD, range)	51.2 (19.1, 16–93)
Sex	
Male	26 (56.5)
Female	20 (43.5)
Triage category	
ATS 1	8 (17.4)
ATS 2	38 (82.6)
Mode of arrival	
Ambulance	26 (56.5)
Helicopter	4 (8.7)
Walked in	15 (32.6)
Transfer	1 (2.2)
ED LOS (h) (mean, SD, range)	6.1 (SD 4.2, 1.3–22.4)
Mechanical ventilation	16 (34.8)
Primary presenting problem	
Trauma	26 (56.5)
Cardiac	7 (15.2)
Toxicological	4 (8.7)
Neurological	4 (8.7)
Systemic infection	1 (2.2)
Gynaecological	1 (2.2)
Burns	1 (2.2)
Respiratory	1 (2.2)

Of 46 participants observed, 30 (65%; Site 1: *n* = 15, Site 2: *n* = 15) were interviewed. Three main themes and eight subthemes emerged from the analysis of the observation and interview data. The three main themes were: (1) being a nurse in the resuscitation area; (2) prioritising pain management; and (3) between being and doing. Themes and sub‐themes are described in the following sub‐sections, with exemplar quotes used to elaborate findings.

### 3.2 Theme 1: Being in the Resuscitation Area

Theme 1 comprises three subthemes which revealed the circumstances that influenced the preparation and practice of emergency nurses to independently care for critically ill patients in the resuscitation area: (i) *being a nurse in the resuscitation area*; (ii) *learning to assess pain*; and (iii) *confidence to manage pain in the critically ill patient*.

#### 3.2.1 Being a Nurse in the Resuscitation Area

This subtheme describes how nurses transitioned into working in the resuscitation area, and challenges experienced in developing critical care knowledge and skills. Emergency nurses working in the resuscitation area were observed to conduct detailed patient assessments, provided symptom management such as pain relief, managed respiratory therapies including mechanical ventilation, communicate complex information to patients, family members, colleagues and visiting inpatient teams, and responded to patient deterioration while simultaneously alerting other ED team members. Transitioning into the resuscitation area was acknowledged as being ‘challenging … [*due to*] the complexity of patients, intensity … compared to working in Acute or Fast Track (Interview, S1‐CNS1)’. During observation, when there were multiple patients in the resuscitation area being cared for by a range of healthcare workers, the management of issues identified during patient assessment did not always proceed in a linear, sequential fashion.

Nurses assess and monitor the patient, give medications, manage multiple demands, and then the inpatient team—who don't always tell you they've charted med[ications] or infusions, or ordered imaging meaning that have to escort patients to CT with little notice. You can't be everywhere, and you can't always be as attentive as you would like or offer one‐on‐one nursing at times. (Interview, S2‐RN12)

Participants frequently reviewed and compared their assessment findings with previous nursing assessments, to identify any new or changing symptoms, signs of deterioration or interventions not yet completed. This also included any new requests for medication administration and diagnostic tests:

To make sure we haven't missed anything, I run back through the primary survey—it's important to know what you need to manage or escalate. (Field note, S2‐RN13)

Patients requiring specialist care were referred by the emergency medical team to inpatient teams to assist in managing the patient's condition. However, while some inpatient teams ‘… talk[ed] to nurses about the patient's progress, plan of care, concerns (Interview, S1‐RN4)’, in many instances nurses had to ‘… flag the inpatient team down before they [*left*] the resuscitation area, to escalate patient care needs (Interview, S2‐RN11)’.

#### 3.2.2 Learning to Assess Pain

This subtheme describes how nurses developed their knowledge and skills in optimising pain management in critically ill patients. At each site, pain management education was provided during orientation to the ED. During observation, participants combined patient reports of pain with physical assessment findings to obtain a detailed picture of the events leading up to injury/illness, its location, nature, associated characteristics, and intensity of the pain:

I've gone through a steep learning curve over the last 2 years … especially pain assessment and using intravenous analgesia, sedation … stronger pain relief medication than we would give on the ward. (Interview, S1‐RN5)

As part of the professional development pathway at both sites, nurses could independently initiate care using local standing orders, that included diagnostics and the initiation/administration of medications to relieve symptoms such as pain. Of the participants interviewed, over a third (*n* = 12, 42%) had successfully completed the Clinical Initiatives Nurse (CIN) training program (NSW Health 2011), and were able to independently initiate analgesia that included paracetamol, ibuprofen, nitrous oxide, morphine and fentanyl, and ‘[*gave nurses*] more of an insight into pain assessment … confidence to discuss pain relief options with the treating team, … but did not include patients in resus [*resuscitation area*] (Interview, S1‐CNS5)’.

While assessing the site of reported pain, participants verbally checked with the patient for any changes in the intensity of pain, observed for flinching or guarding in response to palpating the area, and changes in a patient's facial expression:

You can assess the area they are complaining of having pain in, palpate the skin, move the joint or simply observe and look for signs of bruising or swelling. (Interview, S1‐CNS1)

On probing to clarify observations in the resuscitation area with participants, each described various methods used to measure pain intensity. However, two main approaches were identified: rate the intensity of pain using a numeric value ‘from 0 to 10, 0 being no pain, 10 being the worst pain ever’ (Interview, S2‐RN6) or asking the patient to categorise their level of pain as either ‘… mild, moderate or severe’ (Interview, S2‐RN1).

During observations, intravenous analgesics in addition to sedatives were commonly (75%) administered intravenously to intubated critically ill patients to optimise comfort, relieve anxiety, and facilitate care. Participants were frequently observed to be the first clinicians to detect the presence of pain in critically ill patients. A variety of approaches to detecting pain ranged from monitoring for behavioural or physiological cues:

I rely more on their vital signs, heart rate, blood pressure, or if they're biting on the [endotracheal] tube. (Interview, S2‐RN4)

Less than half (48%) of the patients had some aspect of pain assessment completed. Assessment predominantly focused on identifying the site of pain (*n* = 25, 54%) and clarifying the events that led up to the incident (*n* = 14, 30%). Yet very few (*n* = 7, 15%) critically ill patients were re‐assessed for the presence or changes in pain within a 1 h period. As one participant explained:

You know you need to go back and check [on their pain], but you also juggle other needs—the ventilator, pushing the patient to CT, caring for other patients, answering queries from staff or relatives, even other patients. We probably don't do it very well. (Interview, S1‐RN1)

**FIGURE 1:**
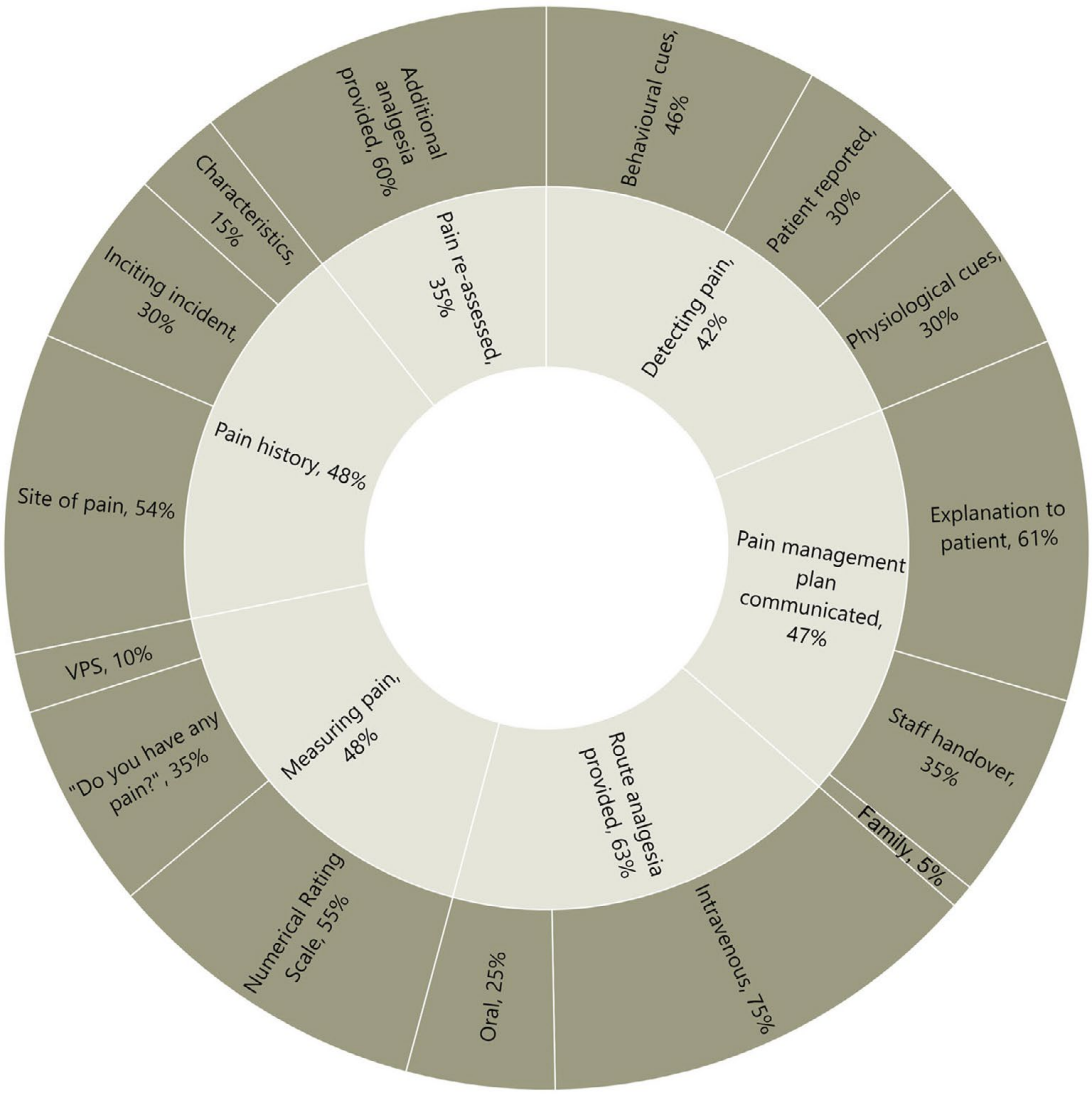
Summary of observed participants pain management activities in the resuscitation area. VPS, Verbal Pain Scale (e.g., mild, moderate, severe).

Across both sites, the majority (63%, *n* = 29) of patients were observed to receive some form of analgesia, although pain history or measurement of pain intensity was less common (48%, *n* = 22), with the focus of any pain history undertaken to identify the site of maximum pain (54%, *n* = 25). In observed instances when patients (*n* = 16) could not self‐report pain, participants monitored for changes in the patient's vital signs (54%) or behaviour (46%). During observation, while the pain management plan was communicated to the patient (*n* = 28, 61%), it was less frequently observed to be discussed at handover (*n* = 16, 35%), or with family members (*n* = 2, 4%) (Figure 1).

Of the 46 patient cases managed by participants, 16 (34.8%) received analgesia in the pre‐hospital setting. The remaining patients (*n* = 30, 65.2%) first received analgesia within 20 min (mean 18.2 min, SD 11.0 min) of arrival in the resuscitation area. While re‐assessment of pain occurred in over half (56.7%) of non‐intubated critically ill patients, re‐assessment of pain in patients (*n* = 16) who were intubated rarely occurred (*n* = 2, 6.6%).

#### 3.2.3 Confidence to Manage Pain in Critically Ill Patients

This subtheme presents the experiences and impact of emergency nurses developing and maintaining confidence in managing acute pain in critically ill patients. For many participants (*n* = 26, 87%), their first experience of managing acute pain in critically ill patients occurred while working in the resuscitation area. Managing acute pain in non‐verbal or unconscious critically ill patients was reported by all participants as ‘intimidating (S1‐CNS1)’ and feeling ‘unconfident (S2‐RN19)’. As one participant with 13 years' experience working in the resuscitation area voiced:

Moving into resus was intimidating, we had no training program back then—you were just thrown in. I remember in my first week in resus, I was caring for an intubated patient … I was trying to work out why the ventilator's high‐pressure alarm kept triggering and it wasn't until a more senior nurse came in … took one look at the patient and quickly bolused morphine then it clicked: the patient was in pain. I felt terrible. (Interview, S1‐CNS1)

During interviews, participants rated their level of confidence on a scale from 0 to 10 (0 least confident, 10 most confident) in managing pain in critically ill patients. Participants indicated having lower levels of confidence in managing acute pain in critically ill patients who were unconscious or non‐verbal, compared to those patients who could self‐report pain (mean 6/10 vs. 9/10 respectively, *Z* = 4.46; *p* < 0.001). All interview participants reported that they would arrange for or provide analgesia if they thought their patient was in pain. Those participants (*n* = 12, 40%) who reported lower levels of confidence (≤ 5) in detecting pain in unconscious or non‐verbal critically ill patients, found identifying or interpreting signs of pain challenging:

Three [out of 10] if I'm honest. It's quite difficult knowing when to give more analgesia, or when to ask a doctor, ‘Can we increase the dosage?’ A lot of the times, nurses shy away from asking the doctor questions and wait to find a more experienced nurse. (Interview, S1‐RN15)

Infrequent exposure to critically ill patients was raised as a major barrier during interviews. Specifically for the many participants (*n* = 14; 46.7%) who undertake other senior roles such as triage and in‐charge, maintaining sufficient time working in the resuscitation area was a concern. As one senior nurses with over 10 years' experience explained:

It's difficult the more senior you become, as you spend less time in resus and more time in coordinating roles. In the last [6 weeks] roster, I had two shifts in resus. I've become acutely aware of losing the ability to do all sorts of resus skills, like caring for ventilated patients … you become less confident. (Interview, S1‐CNS1)

Nurses built upon their pain management knowledge during their careers. Previous and ongoing exposure to managing acute pain in critically ill patients increased nurses' confidence to advocate and administer analgesia. However, reassessment of pain following analgesia was rarely observed. Access and quality of pain relief is reliant upon the knowledge, skills and confidence of the emergency nurse.

### 3.3 Theme 2: Prioritising Pain Management

This theme emerged from observing the behaviours of emergency nurses to prioritise acute pain management. Three sub‐themes highlighted the impacts and challenges in delivering acute pain management in the resuscitation area: (i) *managing the work of resuscitation*; (ii) *minimising delay*; and (iii) *out of sight*.

#### 3.3.1 Managing the Work of Resuscitation

This subtheme presents the challenges of stabilising critically ill patients in this often‐chaotic environment. From observations, the process of assessing, resuscitating, and stabilising patients generated varying levels of workload for the resuscitation nurse and the different members of the resuscitation team:

Emergency nurses are continually busy assessing, monitoring and treating, collecting [pathology] tests, communicating with the patient about their care and the various care team, escalating to various care teams when there are issues, administering medication such as sedation and analgesia, and providing comfort. (Field note, S1‐CNC1)

During observation, participants working in the resuscitation area frequently managed multiple competing care demands for multiple critically ill patients (median 3, range 1–5) with competing complex care needs. When the demand increased, participants either escalated for further nursing or medical assistance, engaged paramedic staff to assist while ED team members assembled, or negotiated as to when they would be able accommodate patient care procedures in the resuscitation area. A typical example of the workload and demand was observed over a 30‐min period involving one senior emergency nurse participant with 18 years' experience (Table 5).

**TABLE 5:** Observed activities across a 30‐min period in the resuscitation area.

Time	Activity
10:01	Ambulance control radios ED to prepare for patient #1, an 84‐year‐old female, GCS 6 requiring airway support and manual ventilation. Participant nurse summons ED medical team using PA system
10:06	Ambulance arrives with patient #1. Participant nurse assists paramedics to transfer the patient #1 to resuscitation bed and takes handover. Summons ED medical team using PA system a second time. Assesses patient using primary survey and commences care. Paramedics provide support
10:18	ED physician arrives and attends patient #1
10:20	A 61‐year‐old male (patient #2) who fell from a ladder (< 1 m), unresponsive, GCS 5, and seizing at scene, is brought into the resuscitation area. Participant nurse uses PA system to request additional nursing and medical assistance
10:21	An additional senior nurse and physician arrives to assist with patient #2
10:25	Nurse‐in‐charge comes into the resuscitation to transfer a stable patient from the acute area (patient #3) needing cardioversion. Participant nurse declines the transfer until further nursing and medical support available
10:30	Neurosurgical team arrive to assess patient #1. Patient now intubated. Neurosurgical team request an urgent CT to be ordered. Participant nurse intervenes, instructing neurosurgical team to order the CT themselves and escort due to workload
10:35	Patient #4, a 36‐year‐old female complaining of abdominal pain and vaginal bleeding for past 2 days now hypotensive at triage, is brought into the resuscitation area. Participant nurse uses PA system to request additional nursing and medical assistance. Triage nurse remains to assist
10:38	ED physician arrives and attends patient #4
10:47	Medical emergency alarm activated at triage—a loud two‐tone alarm sounds throughout the ED. An RN is sent from acute to assist in the resuscitation area
11:00	Cardiology team escorts patient #3 from the acute area. Requests a resuscitation bed. Participant nurse requests procedure be delayed until another resuscitation nurse arrives. Notices consent form has not been completed. Patient returned to acute area

Abbreviation: PA, public address system.

The workload of nurses working in the resuscitation area was variable and unpredictable. The care needs of critically ill patients are complex and can exceed the capacity of nurses working in the resuscitation area. Participants used a range of strategies to increase assistance in order to balance providing timely, safe care and pain relief for patients in the resuscitation area.

#### 3.3.2 Minimising Delay

This subtheme arose from the observed and reported efforts at interview of nurses working to provide timely pain management to critically ill patients. To minimise delay in administering analgesia, nurses used a variety of strategies from negotiation to co‐opting nurses or physicians that entered the resuscitation area to either prescribe, sign out, or administer medication with them:

With only two or three nurses working in resus, things can get delayed. We alert the nurse coordinator, but it can take a while to find a spare nurse to come in and assist—to help catch up. I just grab the first person that walks in, sometimes it's a doctor. (Interview, S2‐CNS4)

While emergency nurses remained vigilant for signs of pain in patients, they experienced multiple challenges in providing timely relief from pain and discomfort. Balancing patient care needs with departmental demands was challenging; a situation made more difficult by some aspects of the resuscitation area layout.

#### 3.3.3 Out of Sight

This next subtheme explores the impact of the resuscitation area layout on communication, visibility, and pain management. In the two study sites, while the resuscitation area was easily accessible from the ambulance bay or triage area, its location and various internal structures limited communication and visibility between clinicians, patients, and the rest of the department. The design of the treatment space and the layout of the resuscitation area impacted on how participants interacted with critically ill patients and provided care, including assessing and managing acute pain:

You can call out and the other nurse can easily hear you. We sometimes pull the middle curtain back if we need to watch one patient whilst working up the patient next to them. (Field note, S1‐RN4)

Participants during interview recounted that staff found this the hardest as they were often unprepared to work in an isolation resuscitation room:

I've definitely noticed, and possible with me as well, there's a reluctance to go in unless you really need to. Once you're in there, trying to get help to pass something into the room, or medication, especially [opioid] analgesia. (Interview, S1‐CNS5)

Emergency nurses experienced multiple challenges in prioritising pain management for critically ill patients. The unpredictability and complexity of critically ill patients managed in a geographically isolated, high acuity environment, reduced the nurse's capacity to detect and respond to the presence of pain, while ensuring other patient care and departmental needs are met. Access to assistance is frequently opportunistic; further limiting the nurses' ability to provide safe, effective care to critically ill patients.

### 3.4 Theme 3: Between Being and Doing

The final theme emerged from observed and reported nurse interactions and behaviours in the resuscitation area to humanise the process of care and the patient's experience of the resuscitation environment. Two subthemes are presented in this section: (i) *being present for the patient* and (ii) the *art of doing*.

#### 3.4.1 Being Present for the Patient

This subtheme was more than the act of completing care tasks, as important as they may have been, but rather being purposefully present for the patient and attentive to the patient's experience of the situation. During interviews, participants acknowledged the importance of building a genuine connection with the patient to increase trust in the care being delivered, and to reduce discomfort and distress, which were recognised as affecting a patient's pain. The following illustrates the voices of all participants:

For many patients, it's the first time they have been to the ED let alone in resus. It can be a highly stressful time, lots of questions, poking and prodding—you can see the stress and embarrassment on their face. You have to be a master of conversation and all‐round interpreter of body language. Getting to know the patient, what they are experiencing, helps guide how you might help, and build trust. (Interview, S1‐CNS6)

As emergency nurses moved around the resuscitation area, they would pause to observe the patient. Participants frequently were observed to look towards the patient to observe their behaviour, that often led to participants initiating a conversation, or small talk, to further explore how patients were coping:

Nurse noticed how quiet the patient was being. The nurse approached the foot of the bed, smiled, and introduced themselves. ‘You look worried. Can I help with anything, or contact a relative or neighbour?’ The patient responded that they were concerned for their wife who gets confused easily when left alone in the house. (Field note, S1‐RN3)

As two experienced participants with over 7 years' experience working in the resuscitation area highlighted, while technical skills are important, connecting with the patients was also important; nurses sought to be present with the patient, not only seek to identify discomfort or distress:

Patients are often scared or anxious. They can be worried about a lot of things from pets at home to what to expect, whilst coping with their injuries. Intubated patients can become agitated—pull lines out or even the [endotracheal] tube. There are many priorities … things that need doing, but if you can spend time with them, listen to their concerns, we can make the patient more comfortable, they feel safer in the [resuscitation area]. (Interview, S2‐RN11)

As observed across the two study sites, how participants positioned themselves relative to the patient, and the degree of line of sight afforded varied, from either sitting next to the patient, around the edges of the resuscitation bay, or just outside the curtain area:

Participant nurse moved mobile workstation and chair to the end of the patient's bed and angled the computer screen to the side to be able to see the patient. ‘Can you see me from here?’ The nurse said. The patient gave a thumbs‐up sign. (Field note, S2‐RN15)

For critically ill patients who were unstable or intubated, participants remained in close proximity to the bedside. All participants were observed to frequently look at the patient's face, constantly monitoring for and reacting to limb movements and signs of deterioration. To maintain connection with patients who were unconscious or intubated, participants were observed to talk to the patient:

The participant continues to talk to the intubated patient—describing what they are doing (wiping blood off the patient's face). The participant then pulls over the computer on wheels and narrates to the patient what they are documenting. (Field note, S2‐RN15)

During one observation period in the resuscitation area, the following was observed between a nurse and a mechanically ventilated critically ill patient:

Participant nurse filled an examination glove with warm water. Ensuring the glove was tied off at the cuff securely, they placed the patient's hand on top, and threaded the fingers of the glove between the fingers of the patient. On probing the nurse behaviour, they explained the importance of patients ‘knowing someone was there … that someone was with them’. (Field note, S2‐CNE1)

At interview, participants supported this sense of being present with the patient by remaining close to them:

It's important that the patient knows whose around them, what's happening to them—even if they are sedated, I still talk to them, make sure things are tidy around them, clean them up. (Interview, S1‐RN10)

The resuscitation area provides care for a diverse patient population who experience varying degrees and types of pain and distress. Nurses focused on caring for the whole patient, not just the chief complaint by actively seeking ways to foster an inter‐relational experience, that is, a nurse–patient relationship that fosters mutual openness and trust to create a caring space for effective moments in an environment that is foreign and chaotic.

#### 3.4.2 The Art of Doing

The *art of doing* emerged from participants' accounts of caring for critically ill patients and observed actions and interactions in the resuscitation area to ameliorate pain using a person‐centred approach. While participants identified and managed potential physical sources of a patient's pain arising from their injuries or as a consequence of necessary life‐saving procedures, all further sought to ameliorate pain through addressing patient anxiety. Participants reported that the resuscitation area is a highly stimulating, chaotic and emotionally charged environment, leading to patients' feeling anxious and stressed; further increasing a patient's sensitivity to acute pain and vice versa:

If a patient is anxious, their sense of pain increases. Similarly, if the patient's pain isn't well controlled, it will make them more anxious—they'll start to worry and stress out. We're very good at getting the analgesia in but working out if the patient is anxious or worried takes time and trust. I'll often talk or inquire about their support systems like family. (Interview, S1‐RN10)

During observation, non‐pharmacological means were used to reduce discomfort and distress to ameliorate patient's pain. Participants aimed to provide person‐centred care, by exploring for and adapting care to include potential cultural, emotional and/or social needs of patients, to reduce anxiety and thereby ameliorate pain. This was particularly noted during one observation, where a participant nurse was assisting in the assessment of a young Muslim woman with suspected head and neck injuries whilst ensuring dignity:

ED physician finishes assessing the patient for overt head and neck trauma and requests a CT head and neck. Participant nurse re‐applies the soft collar to stabilise the patient's neck for transport to CT. ‘I need my head covered’ the patient urged the nurse. The patient looks distressed. The nurse and the patient discuss how best to cover their head given the constraints of the soft collar. The patient agrees for the hijab to be placed loosely over the top of their head and down the sides of her face, to cover as much hair as possible.(Field note, S1‐RN1)

Patients often arrived in the resuscitation area in some degree of pain, partially clothed, and covered in blood, mud and/or street litter. Similarly, at interview, participants shared their approaches to reducing patient anxiety by addressing the emotional and social needs of critically ill patients, while providing emergency nursing care:

It can feel like you leave your dignity at the front door of ED, you're a mess, and sometimes life‐saving care doesn't allow for much modesty. The whole situation is enough to make anyone anxious—a group of random people prodding you, undressing you. You do everything with care and try to be as understanding and calming as possible … holding their hand throughout it all. (Interview, S1‐CNS3)

During interviews, all participants spoke of the important role family members and partners play as significant sources of social support for critically ill patients in reducing their anxiety, stress and worry. Conversely, the absence of family members or concern for a partner's wellbeing could become a source of patient worry and stress. Participants reported using a variety of strategies to ensure connections with family or partners, especially important during the COVID restrictions imposed in NSW which limited visitors in hospitals:

We showed patients how to connect with the hospital's free WIFI network and how to start a Skype chat on their phone. If their mobile phone was flat, we had charging leads. You had to get tech‐savvy. Patients can worry a lot—being able to see or hear their relative really de‐stressed them. (Interview, S2‐CNS3)

Similarly, observed on several occasions were participants working with patients to assist in connecting with relatives or resolve patient's concerns for their relative*s*. Participants were observed to use a range of closed and open questions when working with patients in the resuscitation area, including in the isolation rooms, to connect patients to with family members: ‘Do you want us to contact your daughter to be here with you? (Field note, S1‐RN1)’ or, ‘If you have a mobile phone, I can show you how to Skype or Zoom—they can then see you (Field note, S1‐RN6)’, and alternatively ‘We have an iPad we can use to Skype call your wife—it doesn't cost anything (Field note, S1‐RN10)’.

The following interaction was observed:

Participant nurse documenting patient observations on the computer inside the patient's resuscitation bay, notices a discussion between patient and their wife. The nurse steps in and asks the patient's wife, ‘Your husband [patient] looks worried. Have you had breakfast or need a cup of tea or morning medications?’ The wife explains they rushed out the house when the ambulance came. Nurse arranges for the patient's wife to book into ED, and for a doctor to chart their missed medications. Patient's shoulders visible relax, and the patient thanked the nurse, ‘She's all I have’. (Field note, S2‐CNS4)

However, participants could find it progressively more difficult to provide a person‐centred care approach during times of high demand and increased workload in the resuscitation area. While participants' behaviours remained professional and respectful towards the patient, their ability to spend time with a patient, and deliver care that included the preferences and values of the patient were constrained. As one participant expressed explained during interview:

Resus[*citation*] can become so busy that there isn't any time to spend with the patient. Sometimes all you can do is get as many jobs done, as many tasks completed as possible, and apologies to the patient. Things they might need have to go on a list for later. (Interview, S1‐RN1)

Nurses demonstrated a range of expertise and person‐centred caring behaviours to develop and support an effective nurse–patient relationship; actively seeking ways to be present with the patient and their family members and deliver care that included their preferences and values. However, at times, the rapidly changing environment of the resuscitation area constrained the capability of nurses to pursue actions that they perceived would optimise the well‐being and comfort of the patient.

## 4 Discussion

This is the first known study to explore acute pain management practices of emergency nurses in the resuscitation area. The key study findings indicate that emergency nurses working in the resuscitation area need to be highly experienced and skilled to manage acute pain in undifferentiated critically ill patients. Preparing nurses to work independently in the resuscitation area can, however, be an intimidating experience without access to training and support. Emergency nurses face a variety of challenges in assessing and responding to pain in critically ill patients. This study offers significant insights into clinical practice and patient care. Findings illustrated that the detection and control of acute pain management relied upon the confidence, knowledge, and experience of the emergency nurse allocated to the resuscitation area. Workload and communication factors, along with competing priorities and variation in pain assessment methods, may contribute to inadequate pain management.

Historically, relief from acute pain through the provision of analgesia could only be performed by a physician (Collier 2018). Quality of pain control is now a patient (Pogatzki‐Zahn et al. 2019) and nursing sensitive outcome indicator (Feller et al. 2023). Emergency nurses have readily demonstrated that they can safely initiate a wide range of analgesics to relieve pain to a satisfactory level (Sweity et al. 2022). In our study, emergency nurses actively participated in the assessment and management of acute pain. While focus was directed towards identifying the cause of the pain, participants reported having lower levels of confidence in assessing and managing acute pain in critically ill patients who could not self‐report. Patient self‐reporting is the reference standard measure of pain (Herr et al. 2019). However, owing to sedation, altered consciousness, and endotracheal intubation, many critically ill patients are unable to effectively communicate their pain through self‐report (Devlin et al. 2018).

In this study, critically ill intubated patients were rarely assessed for pain or following administration of analgesia, with nurses more frequently relying on monitoring for changes in vital signs to detect the presence of pain. Yet 40% of critically ill patients managed in ED experience moderate to severe pain (Wu et al. 2022). Despite the fact that pain has been studied in mechanically ventilated patients during the last 20 years (Azevedo‐Santos and DeSantana 2018), about 80% of patients still experience moderate to severe pain (Czernicki et al. 2019). Using vital signs to detect the presence of pain is not supported by the literature. In a recent systematic review examining 30 studies totalling 3469 patients across six continents (Shahiri and Gélinas 2023), vital signs demonstrated poor reliability and validity as an indicator of pain being present or to judge its intensity. Unnoticed and unrelieved pain is one of the main sources of psychological and physiological stress for critically ill patients, which can further result in harmful multisystem consequences (Bhattacharyya et al. 2024). In patients unable to self‐report, behavioural pain assessment tools are required (Herr et al. 2019). However, the use of behavioural pain assessment tools in critically ill adult patients is uncommon in ED (Varndell et al. 2020), potentially delaying access to analgesia and early optimisation of pain control. In areas where behavioural pain assessment tools have been incorporated into the management of acute pain in critically ill patients, they have led to greater precision in the relief of pain, reduced medication side effects, improved communication between clinicians, and improved patient outcomes (Chanques and Gélinas 2022). It is therefore essential that evidence‐based behavioural pain assessment tools are used at the outset in the resuscitation area to guide pain management. The use of behavioural pain assessment tools in conjunction with education may also increase nurse confidence (Grommi et al. 2023).

This study found that nurse confidence in acute pain management in critically ill patients varied. Those without previous critical care experience, access to assistance, or infrequently rostered to work in the resuscitation area due to undertaking other senior roles (e.g., in charge, triage) reported feeling less confident in managing acute pain in intubated critically ill patients. Confidence in detecting and managing acute pain among healthcare professionals is variable (Rababa et al. 2021; Douglass et al. 2009). Use of validated behavioural pain assessment tools coupled with education has been identified to improve nurse confidence and reduce the risk of critically ill patients not receiving analgesia. In a recent systematic review, pain management education reduced the risk of patients not receiving analgesia by 40% (Grommi et al. 2023), with the most effective education strategies utilising audit and feedback, collaborative development of quality indicators and policies (Guion et al. 2018), evidence‐based pain assessment instruments (Varndell et al. 2017b) and external benchmarking (Benditz et al. 2016). As also noted in the literature (Varndell et al. 2020), this study found that emergency nurses predominantly developed pain management knowledge through workplace learning activities and peer support. Emergency nurses have a positive attitude towards administering pain relief, and their pain management practices are strongly influenced by their peers (Varndell et al. 2020). However, the average percentage of theoretical pain management knowledge among emergency nurses is variable, ranging from 40.3% to 76.0% (Ahmadi et al. 2023; Moceri and Drevdahl 2014). While pain management education can improve clinical practice (Innab et al. 2022), there is currently no standardised education programme to support emergency nurses managing acute pain in critically ill undifferentiated patients. Peer support could be leveraged through the implementation of pain management champions (Sampson and Johnson 2023) to further improve the quality of pain control, sustain best pain management practices, and improve the uptake of evidence‐based practice.

This study also offered significant insight into the nurse–patient relationship within the resuscitation area. Nurses were observed to be keenly sensitive to patient discomfort and distress and responded to emotional cues from patients and their relatives. Participants actively sought ways to be present with the patient, altering the workspace area or environment (e.g., turning lights down) to create and maintain a therapeutic nurse–patient connection and to optimise patient cultural safety, dignity and privacy. Nurses also took innovative steps to ensure critically ill patients could communicate with family and carers while in the resuscitation area. Communicating with family and support networks is central to patient comfort and confidence in the care provided (Kydonaki et al. 2020). A nurse's ability to be compassionate, caring and communicative evolves over time and reflects the art of nursing (Palos 2014). Comfort is an expression of nursing art consisting of intentional activities by which discomfort is reduced (Malinowski and Stamler 2002). This study found that emergency nurses were aware of the interconnectedness between discomfort and perception of pain and revealed the many ways nurses sought to decrease discomfort and distress in critically ill patients. Unrelieved anxiety and discomfort negatively impact on how patients can experience pain (Mei et al. 2021). To date, there has been limited research pertaining to the concept of comfort within the context of emergency care.

### 4.1 Strengths and Limitations

While the sample size was appropriate for a descriptive qualitative study, and the data provided a rich description of emergency nurse's perceptions and experiences regarding pain management practices for critically ill intubated patients in the resuscitation area, there are several limitations to consider. The study was conducted in two Australian metropolitan EDs, therefore generalisation of research findings may not be possible to tertiary or rural EDs. Guided by data saturation, this study observed 46 emergency nurses of which 30 agreed to be interviewed of varying years of experience working in the resuscitation area. The experiences and perceptions of these participants may not be entirely reflective of the larger emergency nursing community, thus further research is required. Observations and interviews were conducted by the primary author, but data linkage (i.e., auditability), analysis and forming of themes was reviewed by the entire research team to increase study trustworthiness. The presence of a non‐participant observer may have impacted on a participant's regular practice. Further, nurses self‐reported their interpretation of assessment findings and clinical reasoning process. It may be possible that participants did not verbalise aloud their entire thinking process during observation by the primary researcher. While observations were short in duration to limit the impact on the observed nurse (e.g., fatigue), practice may have been different outside of this time period, and therefore it may reduce the generalisability of findings. This study was conducted during the COVID‐19 pandemic, and the use of face masks and isolation rooms may have obscured what could be observed in the resuscitation area. To limit this, the researcher asked clarifying questions to capture what care had been provided.

### 4.2 Recommendations for Further Research

These findings provide the basis for suggestions for future research examining emergency nursing practices in managing acute pain in critically ill patients. Efforts to increase the implementation of evidence‐based practice such as the use of validated pain assessment instruments are needed to improve pain assessment and decision‐making. Further research is therefore needed to refine and validate appropriate pain assessment instruments within the ED context, which then could inform protocol development to enable emergency nurses to administer and titrate analgesia to the needs of critically ill patients.

Study findings further highlight variation in training and support concerning acute pain management in critically ill patients. Further research is required to identify the best method, outcome measures, length of intervention, and follow‐up in delivering pain management education, including assessing cost and long‐term retention of information. The education approach adopted could also be supported by the development of a pain management champion role in the ED that could assist in disseminating best practice and enable nurses to develop confidence in and a consistent and methodical approach to pain assessment in critically ill patients.

Based on this study's findings, further research is required to examine the concept of comfort and its impact on the patient and their family members. In this study, nurses used a variety of actions and behaviours to reduce patient discomfort and anxiety to improve pain relief. Further research is required to examine the safety, feasibility, and impact of devices mimicking hand holding in intubated critically ill patients in the ED.

## 5 Conclusion

Emergency nurses working in the resuscitation area undertake a highly specialised role; often managing multiple undifferentiated patients with life‐threatening conditions and specifically including the assessment and management of acute pain. This study is the first to examine the perceptions and experiences of emergency nurses assessing and managing acute pain in critically ill patients. Our findings identified that the emergency nurse provided a continuity of patient care, and optimised pain control for critically ill patients. Hence, timely effective pain management in critically ill patients relies upon the knowledge, skills, expertise and confidence of the emergency nurse. However, poor levels of pain management knowledge, varying complex workloads, geographical isolation, communication and lack of evidence‐based tools or protocols increase the risk of poor pain management in critically ill or injured patients. There is an urgent need to develop education programs and guidelines to assist emergency nurses to manage acute pain in the critically ill patient.

## Author Contributions

All the authors made substantial contributions to conception and design or acquisition of data or analysis and interpretation of data; involved in drafting the manuscript or revising it critically for important intellectual content and given final approval of the version to be published. Each author should have participated sufficiently in the work to take public responsibility for appropriate portions of the content. The authors agreed to be accountable for all aspects of the work in ensuring that questions related to the accuracy or integrity of any part of the work are appropriately investigated and resolved. All the authors have agreed on the final version and meet at least one of the following criteria (recommended by the ICMJE [http://www.icmje.org/recommendations/]): (1) substantial contributions to conception and design, acquisition of data, or analysis and interpretation of data and (2) drafting the article or revising it critically for important intellectual content.

## Conflicts of Interest

The authors declare no conflicts of interest.

## Supporting information


Data S1.

Data S2.

## Data Availability

The participants of this study did not give written consent for their data to be shared publicly; supporting data are not available.
