# Supplementary material for: Emergency Nurses' Perceptions and Experiences in Managing Acute Pain in Critically Ill Adult Patients: A Qualitative Study
Source: J Adv Nurs. 2025 May 10;82(1):791–806. doi: 10.1111/jan.17033 (PMC12721928; doi:10.1111/jan.17033)
Supplement: Supplementary file 1 — Data S1. [file JAN-82-791-s002.docx]

**Supplemental File 1: Observation guide**

| **Observation dimension, definition** | **Field note prompts** |
| --- | --- |
| Space, the physical layout | - How is the resuscitation area designed? - What equipment/medication is contained in the resuscitation area? - Where are nurses positioned within the resuscitation area? - How do patients enter/exit the resuscitation area? |
| Actors, range of people involved | - Who is present in the resuscitation area? - Who assesses pain in the resuscitation area? - Who communicates about pain assessment findings and their management? |
| Activity, single actions people undertake | - How is pain assessed in the resuscitation area? - What prompts nurses to assess for pain? - How are nurses trying to improve patient comfort? |
| Events, the sequence of activities carried out | - What barriers or enablers occur in the resuscitation area regarding pain management? - What issues are occurring in and around pain assessment or administration of analgesia? - How frequent is pain assessed? - How frequent is analgesia administered |
| Time, the sequencing of events that occur | - When is pain assessed? - When do nurses escalate about the presence/intensity of patient pain? |
| Goal, the objective/plan that people are trying to accomplish | - What strategies are nurses using to optimise pain control? - How do nurses make patients more comfortable - How are pain management goals communicated amongst nurses/clinicians/patients/family members? |
| Feelings, the emotions felt and expressed between people | - What emotions are communicated between the nurse(s), other clinicians, the patient or family members. - How is relief from pain or discomfort expressed? |
