# Supplementary material for: Emergency Nurses' Perceptions and Experiences in Managing Acute Pain in Critically Ill Adult Patients: A Qualitative Study
Source: J Adv Nurs. 2025 May 10;82(1):791–806. doi: 10.1111/jan.17033 (PMC12721928; doi:10.1111/jan.17033)
Supplement: Supplementary file 2 — Data S2. [file JAN-82-791-s001.docx]

**Supplemental file 2: Interview guide**

This study is about how emergency nurses assess and manage acute pain in critically ill patients cared for in the resuscitation area.

| 1. How are ED nurses involved in managing acute pain in critically ill patients? 2. How do you assess pain in the critically ill patient that you are caring for? 3. Could you give examples of how you manage acute pain in the critically ill patient? | |
| --- | --- |
| 1. How did you develop the knowledge and experience in managing acute pain in critically ill patients? 2. What pain management knowledge do you think an emergency nurse needs to care for a critically ill patient safely? | |
| 1. Out of 10 (1, not at all confident, 10, extremely confident) how confident do you feel in managing acute pain in conscious critically ill patients in ED? | |
| 1. What resources (e.g. staff, policies) are available to support you in managing acute pain critically ill patients? 2. What challenges have you experienced in accessing those resources in the resuscitation area? | |
| 1. In your experience, what makes managing acute pain in critically ill patients easier? 2. In your experience, what makes managing acute pain in critically ill patients more difficult? | |
| 1. Out of 10 (1, not at all confident, 10, extremely confident) how confident do you feel in managing those difficulties? | |
| 1. What patient observations help you determine the adequacy of pain management in an unconscious or intubated critically ill patients you care for? |  |
| 1. Out of 10 (1, not at all confident, 10, extremely confident) how confident do you feel in managing acute pain in unconscious or non-verbal critically ill patients? |  |
| 1. If you thought your critically ill patient was in pain, what pharmacological interventions would you use? 2. If you thought your critically ill patient was in pain, what non-pharmacological interventions would you use? 3. Out of 10 (1, not at all confident, 10, extremely confident) how confident would you be in adjusting analgesia being administered to an unconscious or non-verbal critically ill patients? |  |
